# Evaluation of Risk Factors and Prognostic Outcomes in Patients With Intracerebral Hemorrhage: A Clinical Study From a Tertiary Care Hospital in South India

**DOI:** 10.7759/cureus.90871

**Published:** 2025-08-24

**Authors:** Bhaktavatsalam Peta, Srinivas Rao Mallela

**Affiliations:** 1 Department of Pathophysiology, St. George's University School of Medicine, St. George's, GRD; 2 Department of Internal Medicine, Osmania General Hospital, Hyderabad, IND

**Keywords:** ct brain, glasgow coma scale, hematoma volume, hypertension, intracerebral hemorrhage, prognosis, risk factors, stroke

## Abstract

Background: Spontaneous intracerebral hemorrhage (ICH) is a major cause of stroke-related morbidity and mortality, contributing to a substantial proportion of cases worldwide. Outcomes remain poor despite medical and surgical advances, highlighting the importance of identifying modifiable risk factors and reliable prognostic indicators.

Objective: To evaluate the clinical, radiological, and demographic risk factors associated with spontaneous ICH and identify predictors of prognosis among patients admitted to a tertiary care hospital in South India.

Methodology: A prospective observational study was conducted on 40 patients with confirmed spontaneous ICH admitted to the Emergency Department of Mahatma Gandhi Memorial Hospital, Warangal. Data were collected on demographic variables, clinical presentation, risk factors (e.g., hypertension, alcohol, and smoking), and radiologic parameters (e.g., hematoma site, volume, and Glasgow Coma Scale (GCS) score). Outcomes were measured in terms of survival, neurological recovery, and GCS scores.

Results: Hypertension was the most common risk factor, present in 28 (70%) patients. Alcohol consumption and smoking were also significantly associated with spontaneous ICH. Poor prognostic indicators included low initial GCS score, larger hematoma volume (>30 cm^3^), presence of intraventricular hemorrhage, hydrocephalus, and midline shift. Mortality was higher in patients over 60 years and those with thalamic or brainstem bleeds.

Conclusions: Hypertension remains the primary modifiable risk factor for spontaneous ICH in this population. Initial GCS score, hematoma volume, and intraventricular extension are strong prognostic indicators. Early identification and targeted management of these factors may help improve outcomes.

## Introduction

Spontaneous intracerebral hemorrhage (ICH) is defined as bleeding into the brain parenchyma that occurs spontaneously, without trauma or surgery. It accounts for approximately 10%-15% of all strokes and is associated with the highest mortality among stroke subtypes, with rates ranging from 30% to 50% within the first month of onset [1,2]. The World Health Organization defines stroke as a "rapidly developing clinical sign of focal disturbance of cerebral function, lasting more than 24 hours, with no apparent cause other than vascular origin" [3].

The burden of ICH is disproportionately higher in low- and middle-income countries like India, where access to early diagnosis and advanced neurocritical care is limited. In India, ICH contributes to 13%-18% of all stroke cases, based on limited population-based studies [4]. Hypertension has consistently been identified as the most significant modifiable risk factor for spontaneous ICH [5,6]. Other contributing factors include excessive alcohol intake, cigarette smoking, anticoagulant or antiplatelet therapy, cerebral amyloid angiopathy, vascular malformations, and systemic coagulopathies [7-10].

While many of these risk factors are modifiable, early identification and management are key to reducing the burden of disease. Furthermore, several neuroimaging and clinical markers such as hematoma volume, Glasgow Coma Scale (GCS) score at admission, presence of intraventricular hemorrhage (IVH), midline shift, and hydrocephalus have been reported to correlate with poor outcomes [11,12]. Despite advancements in radiologic diagnostics and neurosurgical interventions, overall functional recovery remains poor in most patients, particularly in those with deep-seated hemorrhages or extensive neurological deficits at presentation [13].

Given the paucity of comprehensive Indian studies focusing on both risk factors and prognostic markers of ICH, this study was designed to evaluate these variables in a cohort of patients admitted with spontaneous ICH at a tertiary care hospital in South India.

## Materials and methods

Study design and setting

This prospective observational study was conducted in the Medical Wards of Mahatma Gandhi Memorial Hospital, a tertiary care teaching facility affiliated with Kakatiya Medical College, Warangal, India. The study period extended from January 2018 to June 2019 and included patients presenting with clinical features suggestive of acute stroke, subsequently confirmed to have spontaneous ICH on neuroimaging.

Participants

A total of 40 consecutive patients, aged 18 years or older, with a diagnosis of spontaneous ICH confirmed by non-contrast computed tomography (CT), were enrolled after meeting the eligibility criteria and providing informed consent. Eligible patients were those admitted within 24 hours of symptom onset.

Patients were excluded if the hemorrhage was traumatic in origin or secondary to an arteriovenous malformation, aneurysm, or brain tumor. Additional exclusion criteria included hemorrhages associated with anticoagulant or thrombolytic therapy given for another indication, as well as subarachnoid, subdural, or epidural hemorrhages.

Data collection

Detailed demographic data, clinical history, and neurological examination findings were recorded using a structured proforma. Risk factors, including hypertension, diabetes mellitus, smoking, alcohol consumption, and a history of previous stroke, were documented. Blood pressure at presentation and prior history of hypertension were specifically noted. Baseline hematological and biochemical investigations, including serum cholesterol levels, were performed for all patients.

Neuroimaging with a non-contrast CT scan was used to assess hematoma location, volume, presence of midline shift, intraventricular extension, and hydrocephalus. Hematoma volume was calculated using the ABC/2 method, where A represented the greatest hemorrhage diameter, B the diameter perpendicular to A, and C the number of CT slices with hemorrhage multiplied by the slice thickness in centimeters.

Outcome assessment

Patients were monitored throughout their hospital stay, and outcomes were assessed based on survival status, changes in neurological function, GCS score at discharge, and the presence of residual neurological deficits.

Statistical analysis

Data were entered into Microsoft Excel and analyzed using SPSS software (IBM Corp., Armonk, NY). Descriptive statistics, including means, standard deviations, and frequency distributions, were calculated for all variables. The chi-square test was used to evaluate categorical variables, while the t-test was applied for comparison of means. A *P*-value of less than 0.05 was considered statistically significant.

## Results

Demographic characteristics

During the study period, 40 patients with CT-confirmed ICH were evaluated for risk factors and prognostic indicators. Twenty patients (50%) were between 50 and 69 years of age, 12 (30%) were aged 70 years or older, and 8 (20% were between 35 and 49 years. The youngest patient was 38 years old, and the oldest was 82 years old. Overall, 32 (80%) patients were over 50 years of age.

There was a male predominance, with 24 men (60%) and 16 women (40%). The mean age for male patients was 61.54 ± 12.61 years, and for female patients, it was 62.06 ± 9.91 years, resulting in an overall mean age of 61.75 ± 11.47 years. There was no statistically significant difference in age distribution between sexes (*P *< 0.890). Similarly, the incidence of ICH did not differ significantly between men and women (*P* < 0.206) (Table 1).

**Table 1:** Distribution of patients with respect to age and sex.

Age (years)	Male		Female		Total	
	No.	%	No.	%	No.	%
18-34	0	0	0	0	0	0
35-49	6	15	2	5	8	20
50-69	12	30	8	20	20	50
> 70	6	15	6	15	12	30
Total	24	60	16	40	40	100

Risk factor analysis

Hypertension was the most prevalent risk factor, present in 28 patients (70%), and its association with ICH was statistically significant (*P* = 0.011). Alcohol consumption was reported in 16 patients (40%), and smoking in 10 patients (25%). Less frequent risk factors included diabetes mellitus in four patients (10%), oral anticoagulant therapy in three patients (7.5%), ischemic heart disease in three patients (7.5%), a family history of cerebrovascular accident in three patients (7.5%), aspirin use in two patients (5%), and chronic kidney disease in two patients (5%; Table 2).

**Table 2:** Distribution of major risk factors in patients with ICH. ICH, intracerebral hemorrhage

Risk factors	Number	Percentage
Hypertension	28	70
Alcohol consumption	16	40
Smoking	10	25
Diabetes mellitus	4	10
Anticoagulation therapy	3	7.5
Presence of ischemic heart disease	3	7.5
Drug abuse (cocaine/amphetamine)	-	-
History of cerebrovascular accident in first-degree relatives	3	7.5
Other drugs: Aspirin	2	5
Presence of renal disease	2	5

Assessment of factors for prognostic outcome

GCS and Outcome

Admission GCS score showed a strong correlation with prognosis. All patients with a GCS score of 3-4 died (100% mortality), and those with scores of 5-8 had an 83.33% mortality rate. In patients with scores of 9-13, the mortality rate was 31.81%, whereas all patients with GCS scores of 14-15 survived. These results indicate that lower admission GCS scores are strongly predictive of poor outcomes (Table 3).

**Table 3:** Glasgow Coma Scale and outcome.

GCS score	Total cases		Alive		Dead	
	No.	%	No.	%	No.	%
3-4	4	10	0	0	4	100
5-8	6	15	1	16.67	5	83.33
9-13	22	55	15	68.19	7	31.81
14-15	8	20	8	100	0	0

Radiological Findings: Site of Hematoma and Outcome

The most common site of hemorrhage was the basal ganglia (17, 42.5%), followed by lobar regions (10, 25%), the thalamus (8, 20%), the pons (3, 7.5%), and the cerebellum (2, 5%). Mortality rates by location were 7 (41.18%) for basal ganglia hemorrhages, 4 (40.00%) for lobar hemorrhages, 3 (37.50%) for thalamic hemorrhages, 1 (33.33%) for pontine hemorrhages, and 1 (50.00%) for cerebellar hemorrhages. No statistically significant difference was found in the outcome with respect to the site of hematoma (*P* < 0.997). Although cerebellar hematomas exhibited the highest mortality rate (50%), this difference was not statistically significant. The elevated mortality in cerebellar hemorrhage cases may be attributed to their posterior fossa location, which is in close proximity to vital brainstem centers (Table 4).

**Table 4:** Site of hematoma and outcome.

Site of hematoma	Total cases		Alive		Dead	
	No.	%	No.	%	No.	%
Basal ganglia	17	42.5	10	58.82	7	41.18
Thalamus	8	20	5	62.5	3	37.5
Lobar	10	25	6	60	4	40
Cerebellar	2	5	1	50	1	50
Pons	3	7.5	2	66.67	1	33.33
Total	40	100	24	60	16	40

Hematoma Volume and Outcome

Hematoma volume was a significant prognostic factor. Patients with hematoma volumes greater than 30 cm³ 35% (14) had a mortality rate of 78.57% (11), compared to 19.23% (5) in those with volumes less than 30 cm³ (26, 65%). This difference was statistically significant (*P* < 0.001) (Table 5).

**Table 5:** Hematoma volume and outcome.

Volume	Total		Alive		Dead	
	No.	%	No.	%	No.	%
>30 cm^3^	14	35	3	21.43	11	78.57
<30 cm^3^	26	65	21	80.77	5	19.23
Total	40	100	24	60	16	40

Midline Shift and Intraventricular Extension

Midline shift was observed in 14 patients (35%), of whom 10 (71.42%) died, a statistically significant association with poor prognosis (*P* < 0.006). Intraventricular extension was present in 16 patients (40%), and mortality in this subgroup was 75%, also showing a highly significant association with poor outcomes (*P *< 0.001) (Tables 6-7).

**Table 6:** Midline shift and outcome.

Midline shift	Total		Alive		Dead	
	No.	%	No.	%	No.	%
Present	14	35	4	28.58	10	71.42
Absent	26	65	20	76.92	6	23.07
Total	40	100	24	60	16	40

**Table 7:** Intraventricular extension of hemorrhage and outcome.

Intraventricular extension of hemorrhage	Total		Alive		Dead	
	No.	%	No.	%	No.	%
Present	16	40	4	25	12	75
Absent	24	60	20	83.33	4	16.67
Total	40	100	24	60	16	40

## Discussion

This study reaffirmed that hypertension is the predominant and most modifiable risk factor for spontaneous ICH, present in 70% of our patient cohort. This finding aligns with large-scale epidemiological data, which have consistently demonstrated hypertension as the leading contributor to spontaneous ICH, accounting for approximately 60%-80% of cases [5,6].

A significant subset of our patients also reported alcohol consumption (40%) and smoking (25%), both of which have been linked to increased ICH risk in prior studies. Zeng et al. found an independent association between alcohol intake and ICH, irrespective of hypertension status [8], while studies by Tanaka et al. and Kagan et al. documented an increased risk of hemorrhagic stroke among smokers and alcohol consumers, particularly in Japanese populations [9,10]. Furthermore, low serum cholesterol levels have been associated with increased ICH risk. Levels below 160 mg/dL have been linked to vessel fragility and increased hemorrhagic potential, as observed in the Honolulu Heart Program and subsequent studies [10,11].

In our study, GCS at admission emerged as a critical prognostic indicator. Patients with GCS ≤ 8 had mortality rates exceeding 80%, while those with GCS ≥ 14 had 100% survival. This is in line with Broderick et al., who reported that a GCS score <9 was associated with a 90% 30-day mortality rate [1]. The importance of initial GCS has since been incorporated into prognostic tools such as the ICH score [12].

Hematoma volume also demonstrated a strong correlation with outcome. Volumes >30 cm^3^ were associated with 80% mortality in our cohort, echoing Kazui et al., who found that early hematoma expansion, particularly in the first three hours, was significantly associated with clinical worsening [13]. Larger hematomas increase intracranial pressure (ICP) and compress surrounding brain structures, worsening both morbidity and mortality.

There was no statistically significant difference in outcomes with respect to the site of hematoma (*P* = 0.997, NS). Although cerebellar hematomas exhibited the highest mortality rate (50%), this difference was not statistically significant. The elevated mortality in cerebellar hemorrhage cases may be attributed to their posterior fossa location, which is in close proximity to vital brainstem centers.

The anatomical location of the hemorrhage did not significantly impact outcomes. Although mortality appeared higher in cases of cerebellar hemorrhage, this was not statistically significant and may be related to the posterior fossa's proximity to critical brainstem centers. This pattern aligns with existing literature indicating that deep-seated hemorrhages, particularly those involving the brainstem, are associated with poorer prognoses due to their closeness to vital structures and limited options for surgical intervention [14,15].

Our findings also highlight the negative prognostic implications of intraventricular extension and midline shift. The presence of IVH was associated with 75% mortality in our study, reinforcing prior reports that IVH contributes to obstructive hydrocephalus, increased ICP, and worse clinical outcomes [16]. Midline shift, a radiological indicator of mass effect and raised ICP, also significantly correlated with mortality (70%). Previous neurosurgical studies support that midline shift is associated with poor outcomes and guides urgency for surgical decompression [17].

Advanced age was another significant predictor of poor outcome, particularly in patients over 60 years. This is supported by epidemiological data from both Western and Indian cohorts that showed increased stroke-related mortality with advancing age [4,5,18].

The findings should be interpreted in light of certain limitations. The study was conducted in a single tertiary care hospital with a relatively small sample size, which may limit the generalizability of the results. Long-term functional outcomes were not assessed, and follow-up beyond hospital discharge was not performed. In addition, some potentially relevant variables, such as detailed medication history and socioeconomic factors, were not evaluated. Future multicenter studies with larger cohorts and extended follow-up periods are warranted to validate these findings.

## Conclusions

Spontaneous ICH continues to pose a major public health challenge due to its high mortality and morbidity. This study highlights that hypertension is the most prevalent and modifiable risk factor in the local population, and underscores the critical prognostic value of initial GCS score, hematoma volume, intraventricular extension, and midline shift on imaging.

Early identification of these parameters may help clinicians stratify patients based on risk, personalize treatment, and potentially improve outcomes. Public health efforts to promote blood pressure control and reduce modifiable risk factors such as alcohol consumption and smoking may have a substantial impact in reducing the incidence and severity of ICH.
